# Multivalent dendritic polyglycerolamine with arginine and histidine end groups for efficient siRNA transfection

**DOI:** 10.3762/bjoc.11.86

**Published:** 2015-05-13

**Authors:** Fatemeh Sheikhi Mehrabadi, Hanxiang Zeng, Mark Johnson, Cathleen Schlesener, Zhibin Guan, Rainer Haag

**Affiliations:** 1 Institut für Chemie und Biochemie, Freie Universität Berlin, Takustrasse 3, 14195 Berlin, Germany; 2 Department of Chemistry, University of California, 1102 Natural Sciences 2, Irvine, California 92697-2025, USA

**Keywords:** arginine, dendritic polyglycerolamine, histidine, multivalent vector, siRNA delivery

## Abstract

The success of siRNA-based therapeutics highly depends on a safe and efficient delivery of siRNA into the cytosol. In this study, we post-modified the primary amines on dendritic polyglycerolamine (dPG-NH_2_) with different ratios of two relevant amino acids, namely, arginine (Arg) and histidine (His). To investigate the effects from introducing Arg and His to dPG, the resulting polyplexes of amino acid functionalized dPG-NH_2_s (AAdPGs)/siRNA were evaluated regarding cytotoxicity, transfection efficiency, and cellular uptake. Among AAdPGs, an optimal vector with (1:3) Arg to His ratio, showed efficient siRNA transfection with minimal cytotoxicity (cell viability ≥ 90%) in NIH 3T3 cells line. We also demonstrated that the cytotoxicity of dPG-NH_2_ decreased as a result of amino acid functionalization. While the incorporation of both cationic (Arg) and pH-responsive residues (His) are important for safe and efficient siRNA transfection, this study indicates that AAdPGs containing higher degrees of His display lower cytotoxicity and more efficient endosomal escape.

## Introduction

Since the discovery of RNA interference (RNAi) and awareness of its role in posttranscriptional gene silencing, tremendous efforts and capital have been devoted to the development of therapeutics based on this pathway [1]. So far, there are at least 22 RNAi-based drugs in clinical trials and many more are being developed [1]. Although a direct delivery of “naked” siRNA or chemically modified oligonucleotides [2] has been studied, delivery vectors are typically required for efficient siRNA delivery in vivo due to unmodified siRNA’s low stability towards endogenous enzymes, poor cellular uptake, and its immunogenic potential [3].

Among the different polymeric vectors, polycationic dendrimers and related structures have found wide application in gene/siRNA delivery [4]. This is because the synthesis of dendrimers and dendritic polymers under controlled conditions results in defined structures with low dispersity. Moreover, the tree-like structure of such polymers provides multivalent positions for functionalization and interaction with DNA/siRNA.

Dendritic polyglycerol (dPG) can be synthesized on a kilogram scale by a one-step, ring-opening polymerization of glycidol with controllable sizes and degrees of branching [5]. Additionally, dPG has multiple groups for further functionalization, high chemical stability, and good biocompatibility in vitro and in vivo [6–8]. All these characteristics make dPG an ideal scaffold for a broad range of applications from anti fouling [9] to biomedical purposes [6] such as anti-inflammatory [10] and anticancer therapy [11–12].

Previously a number of cationic polymers like chitosan [13–15], PEI [16], and PAMAM [17] have been post-modified with histidine (His) or arginine (Arg) groups. The introduction of histidine groups has been beneficial for improving the endosomal release properties [18], and conjugation of arginine groups has enhanced the transfection efficiency of cationic carriers [19–20]. Since the incorporation of either amino acid alone can improve siRNA transfection, we hypothesized that functionalization with both Arg and His may have a synergistic effect on siRNA transfection. Moreover, the biocompatible nature of the amino acids can possibly decrease the cytotoxicity of the resulting vectors. Furthermore, Arg and His groups interact in histones, as natural DNA binding proteins, via their positive residues with the negative phosphates groups of the DNA [21]. Here, we chose dendritic polyglycerolamine (dPG-NH_2_) with moderate amine loading (50% of all hydroxy groups on a 10 kDa dPG core) and introduced both amino acids via amide coupling to mimic DNA histones interactions.

In a recent study, our group demonstrated the potential of dPG-NH_2_ with high amine loading (≥90%) for siRNA delivery in vivo [22]. Moreover, it has been shown that dPG-NH_2_ 90% is able to efficiently downregulate the formation of several proteins in vitro [23]. In spite of its high efficiency, the therapeutic window of dPG-NH_2_ 90% is small and the cytotoxicity increases at higher concentrations which limits its further application. Here, we compare the potential of multivalent amino acid functionalized dPGs (AAdPGs), for siRNA transfection with dPG-NH_2_ 90%. The initial in vitro results indicated that AAdPGs were capable of mediating efficient siRNA delivery to NIH 3T3 cells and induced comparable gene silencing to both dPG-NH_2_ 90% and lipofectamine RNAiMAX. In comparison with dPG-NH_2_ 90%, the new vectors showed reduced cytotoxicity and enhanced siRNA binding.

## Results and Discussion

### Functionalization of dPG-NH_2_ with arginine and histidine

**Scheme 1:**
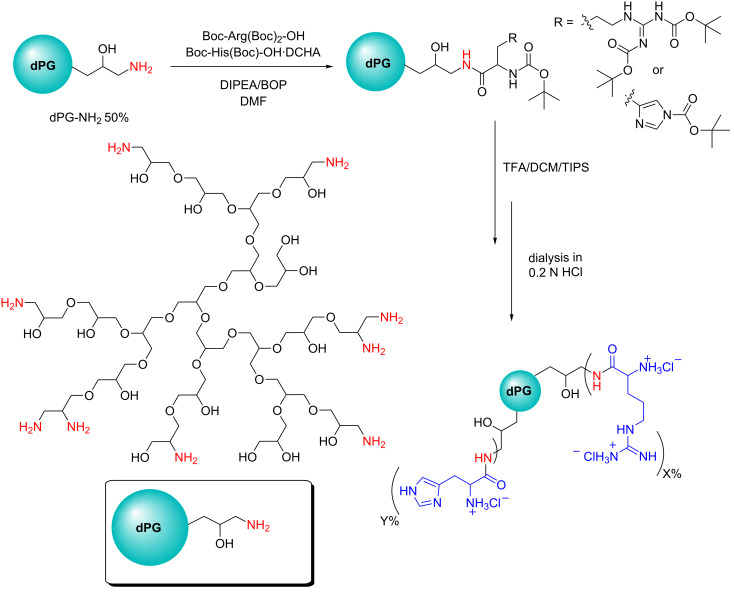
Synthesis of multivalent arginine and histidine functionalized dPG-NH_2_ 50%. The depicted dPG-NH_2_ represents only a small idealized fragment of a 10 kDa molecule.

Amino acids have been implemented for the improvement of gene/siRNA transfection using various strategies. Beside peptide dendrimers [24–25], another strategy is to functionalize the periphery groups on cationic vectors such as PLL [26], PEI [16], and PAMAM [19]. In the current study, ≈50% of all hydroxy groups on dPG (*M*_n_ = 8.4 kDa, PDI = 1.7) were converted to amino groups according to an earlier published procedure (Scheme S1, Supporting Information File 1) [27]. The high density of amines on dPG facilitates the introduction of groups like amino acids by feasible strategies like amide coupling. Here, we coupled both Arg and His groups in different ratios to dPG-NH_2_ via the latter strategy (Scheme 1). By introducing Arg on the dendritic scaffold, this group can serve as a complexing agent and the surplus guanidium groups with high affinity to phosphate groups can interact with the cell membrane and improve the cellular uptake [28]. Additionally, the histidine groups can facilitate tackling the endosomal release problem by improving the polyplexes’s buffering capacity [18]. Moreover, arginine and histidine groups can form intermolecular hydrogen bonds with cell surface phosphate groups. These interactions can induce cellular uptake of AAdPG polyplexes. Therefore, four cationic vectors were prepared by Arg and His functionalization of the dPG scaffold. The list of all synthesized samples is presented in Table 1. The samples were named based on their degree of Arg and/or His functionalization on the polymeric backbone (dPG). The functionalization degree for each polymer was determined by comparing the peak integral of either the methylene groups of arginine in high field or the imidazole ring of histidine in the aromatic area (7.2–8.7 ppm) with the assignable dPG backbone signal (Supporting Information File 1).

### Variable composition of arginine and histidine on dPG-NH_2_ 50%

To investigate the effect from introducing both His and Arg to dPG backbone on transfection efficiency, cytotoxicity, and cellular uptake, two vectors were synthesized with equal (dPG-13Arg13His) and different (dPG-8Arg30His) composition ratios of both amino acids. Moreover, two further vectors with either Arg (dPG-13Arg) or His (dPG-13His) were prepared to examine the effect of each amino acid alone. The summary of all dPG-based vectors is shown in Table 1.

**Table 1:** Summary of AAdPG vectors and their corresponding polyplex characterization.

Compound	Zeta potential (mV)^a^	diameter (nm)^b^	PDI^c^	(Arg) %^d^	(His) %^d^	Arg:His

dPG-NH_2_ 50%	10.0 ± 0.2	124.1 ± 0.7	0.07	–	–	–
dPG-13Arg13His	10.9 ± 0.8	97.17 ± 0.87	0.13	13	13	1:1
dPG-13Arg	10.6 ± 0.9	60.04 ± 1.2	0.18	13	–	–
dPG-13His	10.3 ± 0.3	70.23 ± 0.8	0.17	–	13	–
dPG-8Arg30His	11.0 ± 0.9	104.9 ± 0.45	0.18	8	30	~1:3

^a^ζ were measured at pH 7.4; ^b^intensity distributions are reported; ^c^PDI of polyplexes were determined by DLS; ^d^degree of functionalization on dPG which were determined by ^1^H NMR spectroscopy.

### siRNA Binding

**Figure 1:**
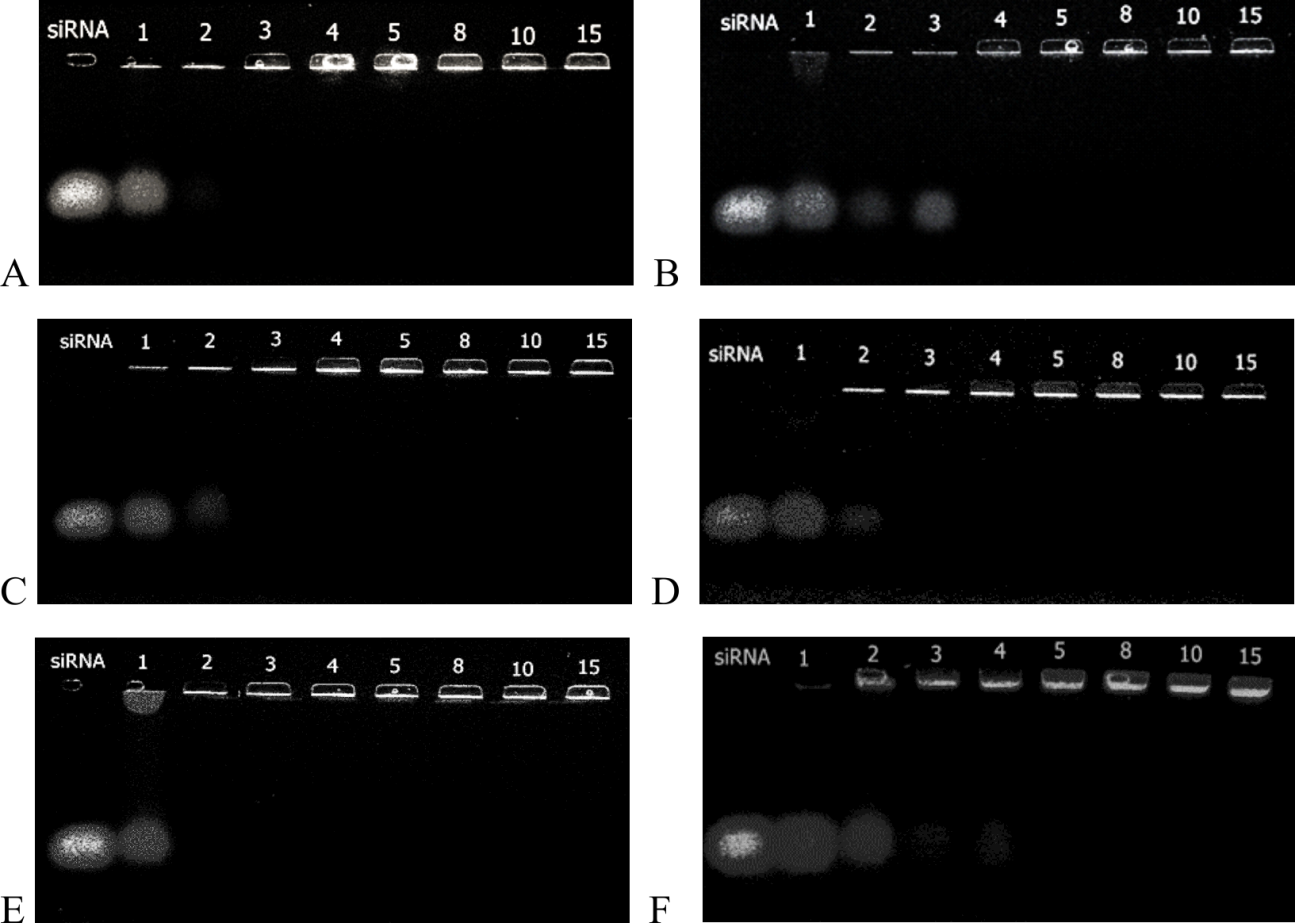
Agarose gel electrophoresis retardation assay of AAdPGs/siRNA polyplexes. (A) dPG-13Arg13His, (B) dPG-13Arg, (C) dPG-13His, (D) dPG-8Arg30His, (E) dPG-NH_2_ 50%, and (F) dPG-NH_2_ 90%. Naked siRNA always appears in the first lane. The numbers on the top of each lane correspond to the different N/P ratios.

The ability of AAdPGs to form complexes with siRNA was examined by agarose gel electrophoresis retardation assay. The electrophoretic mobility of the siRNA should have been reduced or completely eliminated as a result of complexation with AAdPGs. As shown in Figure 1, all AAdPGs were able to neutralize the negative charge of the siRNA and effectively retard it at N/P ratios between 2 to 4. The binding capacity of all vectors was slightly different from each other. The results of this assay clearly display that all synthesized vectors were able to form polyplexes with siRNA at low N/P ratios. Moreover, the complex formation ability of the new vectors is comparable with dPG-NH_2_ 50% and 90%.

### Average particle size and surface charges of AAdPG/siRNA polyplexes

**Figure 2:**
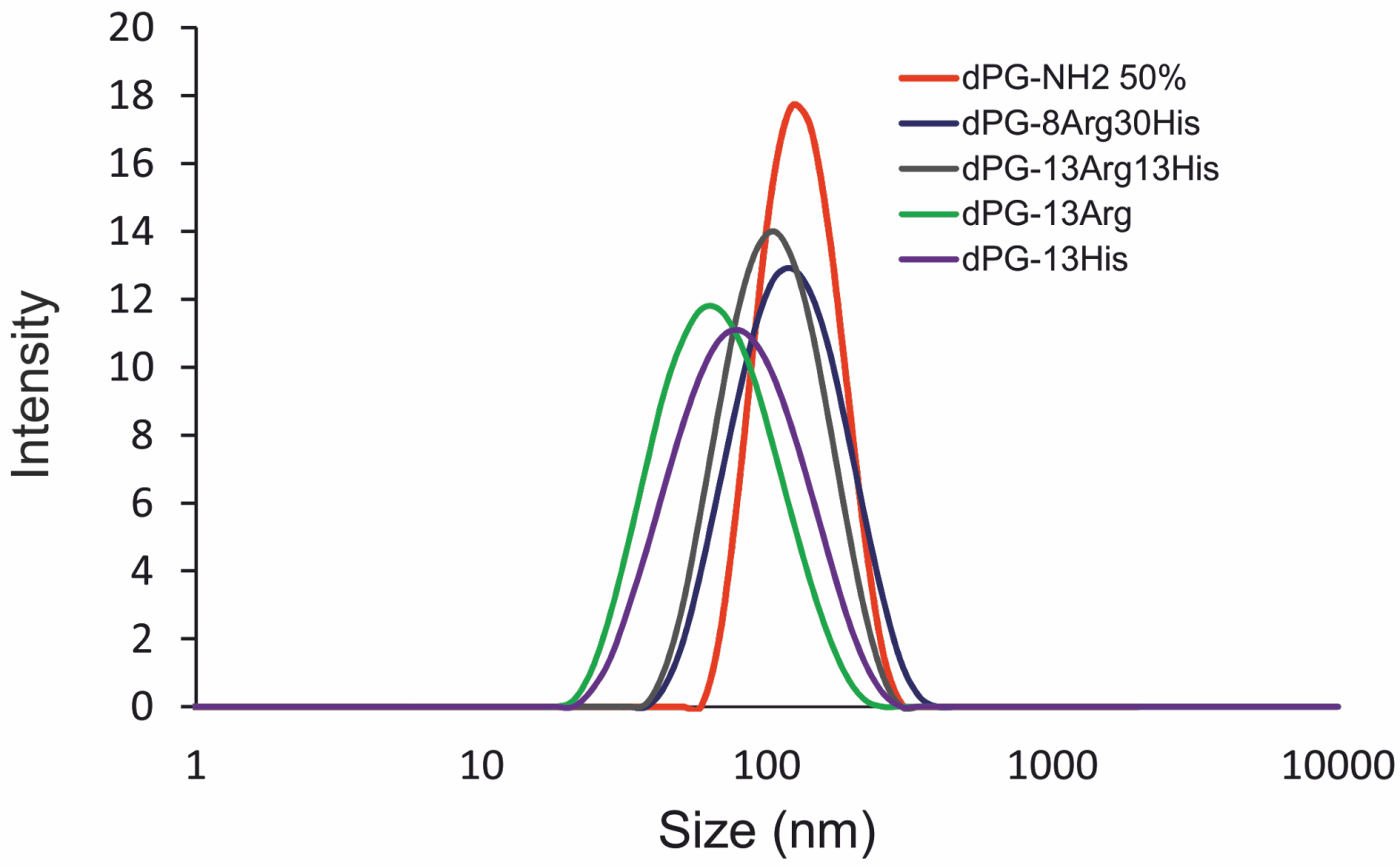
Size measurements of dPG-NH_2_ 50% and AAdPGs/siRNA complexes. Intensity distributions of all polyplexes are depicted.

The appropriate particle size and surface charge are critical characteristics of nanoplexes for efficient transfection [29]. Physicochemical characterization of AAdPG/siRNA polyplexes was conducted using dynamic light scattering (DLS). Figure 2 shows the size distribution of dPG polyplexes (at N/P ratio 10). The average size of all nanoparticles ranges from 60–100 nm. In general, the AAdPG/siRNA polyplexes were smaller than the corresponding dPG-NH_2_ 50%/siRNA polyplexes. Moreover, AAdPG complexes have a broader distribution of the final nanoparticles. The size of dPG-13Arg and dPG-13His complexes was slightly smaller than the other dPG-based vectors. The surface charge of the final nanoparticles was comparable to the corresponding complexes of siRNA and dPG-NH_2_ 50% with terminal primary amines and about 10 mV. The positive charge of the polyplexes is a further indication of efficient siRNA complexation by AAdPGs. The results for the size and zeta potential measurements of all vectors are summarized in Table 1.

### Cell viability assay

**Figure 3:**
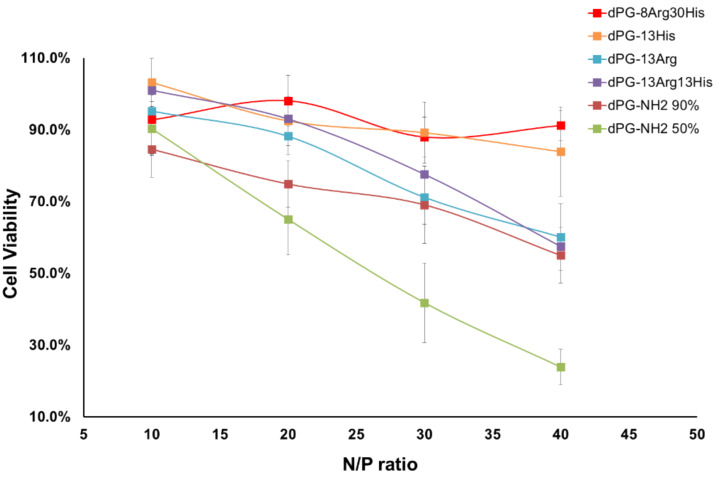
The result of MTT assay on a NIH 3T3 cell line transfected with AAdPG, dPG-NH_2_ 50%, and 90%/siRNA polyplexes at different N/P ratios with 100 nM siRNA concentration.

**Figure 4:**
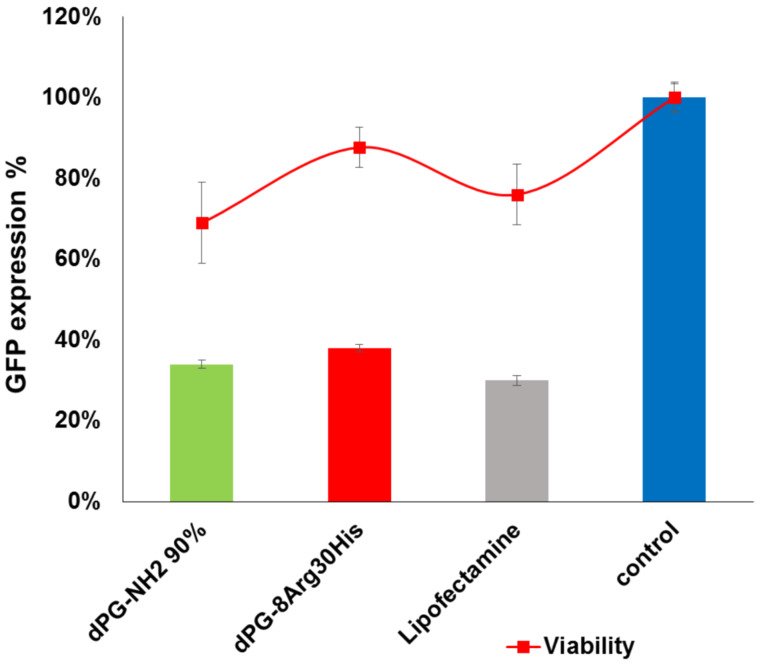
Cell viability versus transfection efficiency of dPG-8Arg30His and dPG-NH_2_ 90% at N/P ratio 30.

The cytotoxicity of cationic polymers is mainly attributed to the interactions of these polymers with the cell membrane and depends on multiple factors such as molecular weight, the nature of the polymer surface, and its charge density [30]. The results of the in vitro MTT assays on the NIH 3T3 cell line for cytotoxicity evaluation of AAdPG polyplexes are shown in Figure 3. These results were compared with dPG-NH_2_ 50% as a backbone and dPG-NH_2_ 90%. Generally, these data indicates that cytotoxicity of the final polyplexes is reduced by functionalization of dPG-NH_2_ 50% with Arg and His. Moreover, decreasing the percentage of arginine on a dendritic scaffold improved the cytotoxicity of the nanoplexes. Replacing the primary amines on dPG-NH_2_ with histidine groups would possibly decrease the density of positive charge on dPG and increase cell viability. The best cytotoxicity profile was observed for dPG-8Arg30His with no considerable cytotoxicity (cell viability ≥ 90%) up to N/P ratio 40 (Figure 3). We further compared the cytotoxicity of dPG-8Arg30His with dPG-NH_2_ 90% at N/P ratio 30 where the efficiencies of both vectors were comparable. Overall, these results demonstrated that dPG-8Arg30His is a safer vector compared to dPG-NH_2_ 90% (Figure 4).

### In vitro transfection assay

**Figure 5:**
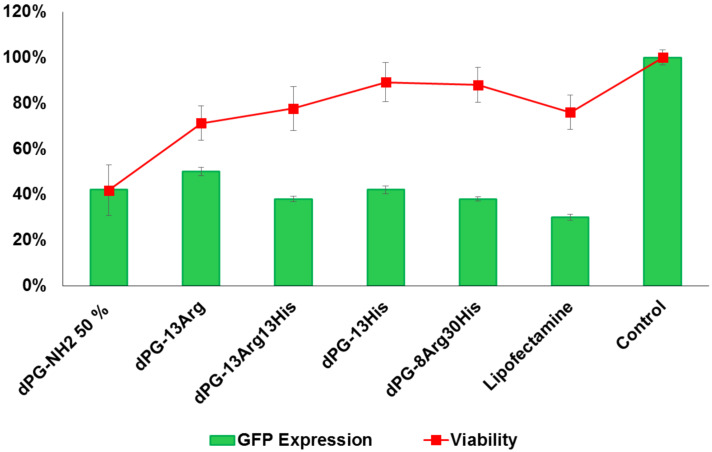
Summary of transfection results versus viability of AAdPGs with various Arg and His composition ratio at N/P ratio 30.

The transfection efficiency of the AAdPGs was assessed in GFP expressing NIH 3T3 cells (Figure 5). In general, the results indicate that post-modification of the dendritic scaffold with Arg and His improves the efficiency of siRNA transfection. The most efficient vector in the knockdown of GFP (down regulation of GFP expression to 38%) was obtained by converting almost all primary amines on dPG to Arg and His with a 1:3 ratio. Moreover, by comparing the knockdown efficiency of dPG-13Arg (without any histidine functionality) with all the other vectors containing histidine, the critical role of histidine as a buffering agent in enhancing transfection efficiency was determined. Furthermore, we compared the result of our best vector, dPG-8Arg30His, in terms of transfection with dPG-NH_2_ 90%. These results indicate that dPG-8Arg30His (at N/P ratio 30) is as potent as dPG-NH_2_ 90% in GFP knockdown while maintaining its low cytotoxicity (Figure 4).

### Cellular uptake and confocal microscopy

**Figure 6:**
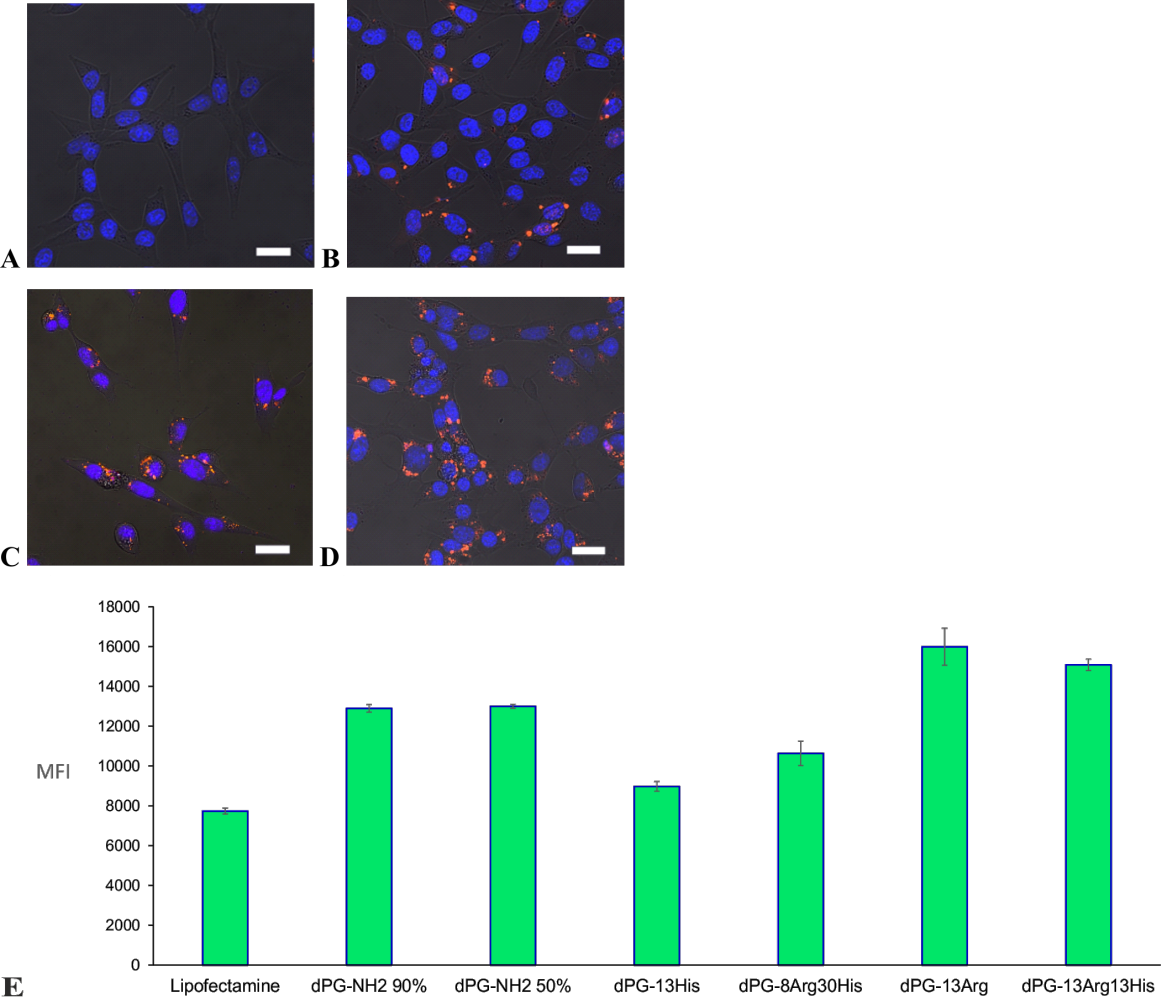
Confocal images of NIH 3T3 cells treated with Cy3-siRNA/vector complexes: (A) naked siRNA, (B) lipofectamine, (C) dPG-13His, (D) dPG-13Arg, and (E) mean Cy-3 fluorescence intensity of 3T3 cells treated with Cy3-siRNA/vector complexes assessed by FACS.

The cellular uptake and localization of fluorescently labeled siRNA/AAdPG complexes were quantified using flow cytometry and confocal microscopy (Figure 6). By comparing the cellular uptake of dPG-NH_2_ functionalized solely with either histidine or arginine, for example, dPG-13Arg, one can clearly see that Arg functionalization improved cellular uptake of both dPG-NH_2_s. These results are in agreement with several studies where the transmembrane function of arginine-rich peptides was demonstrated [31–32]. Interestingly, there is a reverse effect with respect to cellular uptake after functionalization of dPG-NH_2_ with histidine. Notably, dPG-NH_2_s have shown a higher cellular uptake than lipofectamine which is most probably due to their high positive surface charge. These results in combination with transfection efficiency data suggest that the higher transfection efficiency of histidine-functionalized vectors is presumably due to their improved endosomal release.

## Conclusion

We successfully post-modified dPG-NH_2_ with variable ratios of Arg and His as mimicry of natural histones to afford safe and efficient siRNA transfection. At certain ratios of Arg to His (1:3) a multivalent cationic vector was obtained with comparable transfection efficiency to lipofectamine (down regulation of GFP expression to 37% at N/P ratio 40) and marginal cytotoxicity (cell viability ≥ 90% at N/P ratio 40). The efficiency of this new vector is comparable to our well-studied vector, dPG-NH_2_ 90%. Post modification of dPG-NH_2_ with Arg and His did not dramatically affect the physicochemical properties (particle size and zeta potential) of the resulting vectors and their nanoplexes but notably improved cell viability. This can be attributed to the steric congestion around the amine groups and more biocompatible surface functionalities after amino acid functionalization of dPG-NH_2_. Compared to arginine, the introduction of histidine more effectively reduced the cytotoxicity and mediated an efficient endosomal escape. Moreover, by comparing the result of cellular uptake with transfection efficiencies, one can conclude that enhanced cellular uptake does not guarantee by itself efficient siRNA transfection and that incorporation of endosomal releasing groups like histidine seems to play a more crucial role in efficient transfection as compared to arginine.

## Experimental

### Materials

All chemicals and reagents were used as received from the suppliers without further purification. Protected amino acids and coupling reagents were purchased from abcr GmbH (Karlsruhe, Germany). GelRed^TM^ siRNA stain was purchased from VWR (Radnor, PA). All cell culture media and fetal bovine serum (FBS) was purchased from Invitrogen (Carlsbad, CA). All siRNA used in this study was purchased from Ambion (Carlsbad, CA) with Silencer^®^ Select negative control siRNA and Silencer^®^Cy™-3 labeled Negative Control siRNA used for control and cellular uptake studies, respectively. Unmodified Silencer^®^ series siRNA was used for GFP silencing experiments with the following sequence: sense 5’-CAAGCUGACCCUGAAGUUCTT-3’ and antisense 5’-GAACUUCAGGGUCAGCUUGCC-3’. All water used in the biological experiments was nanopure water obtained from Barnstead Nanopure Diamond (Waltham, MA). Both unmodified and engineered NIH 3T3 cells expressing green fluorescence protein (GFP) were kindly provided by Professor Young Jik Kwon (Department of Chemical Engineering, UC Irvine, CA).

### Functionalization of dPG-NH_2_ with arginine (Arg) and histidine (His)

dPG (*M*_n_ = 8.4 kDa, PDI = 1.7) was prepared according to a published procedure [33]. Fifty percent of all (~110) hydroxy groups on dendritic polyglycerol were functionalized with amino groups in a three-step protocol [27]. Briefly, the transformation was started with the mesylation of the hydroxy groups on dPG. In the next step, the mesylated polyglycerol was converted to polyglycerolazide. In the last step, azide functionalities (N_3_) were reduced to primary amines (-NH_2_) via Staudinger reduction (Scheme S1 in Supporting Information File 1). For coupling both amino acids Arg and His to the dendritic backbone, a solution of dPG-NH_2_, 30 mg (0.20 mmol of amines) in methanol, was dried carefully under high vacuum. The concentrated solution was then diluted in 1.5 mL DMSO. The solution of dPG-NH_2_ in DMSO was left under vacuum for 30 min in order to remove methanol residues. Boc-protected histidine and arginine were added to the solution of dPG-NH_2_ in specific molar ratios. 1.2 Equivalents of BOP and DIPEA with respect to the amino groups were added to the reaction subsequently. The reaction mixture was stirred at room temperature overnight. This mixture was then transferred directly into a dialysis tube of 1000 MWCO and dialyzed in methanol for 2 days. After removing methanol on a rotary evaporator completely, the reaction mixture was treated with a mixture of TFA/DCM/TIPS. The reaction was left running overnight to complete the deprotection. After the deprotection step, dialysis in 0.2 N solution of HCl for two days resulted in the formation of products as chloride salt which were obtained as pale yellow to brown solids by lyophilization. Noteworthy that each dPG unit (10 kDa) has is about 100 hydroxy groups and therefore the functionalization percentages always corresponds approximately to the same number of functional groups per dPG. For example, dPG-NH_2_ 50% has about 50 NH_2_ groups per polymer unit. The amino acid functionalization percentage of each polymer was defined using ^1^H NMR analysis. ^1^H NMR (400 MHz, D_2_O) dPG-13Arg13His: δ = 1.6 (s, NHCH_2_C*H_2_*CH_2_CH, 2H), 1.9 (s, NHCH_2_CH_2_C*H_2_*CH, 2H), 3–4.5 (m, dPG backbone, NHC*H_2_*CH_2_CH_2_CH and NHCH_2_CH_2_CH_2_C*H*NH_2_CO of arginine groups, NH_2_COC*H*CH_2_C and NH_2_COCHC*H_2_*C of histidine groups), 7.4 (s, C*H*NHCHN, 1H of imidazole groups) and 8.7 (s, CHNHC*H*N, 1H of imidazole groups) ppm. dPG-13Arg: δ = 1.6 (s, NHCH_2_C*H_2_*CH_2_CH, 2H), 1.9 (s, NHCH_2_CH_2_C*H_2_*CH, 2H), 3–4.5 (m, dPG backbone, NHC*H_2_*CH_2_CH_2_CH and NHCH_2_CH_2_CH_2_C*H*NH_2_CO of arginine groups) ppm. dPG-13His: δ = 3–4.5 (m, dPG backbone, NH_2_COC*H*CH_2_C and NH_2_COCHC*H_2_*C of histidine groups), 7.4 (s, C*H*NHCHN, 1H of imidazole groups) and 8.7 (s, CHNHC*H*N, 1H of imidazole groups) ppm. dPG-8Arg30His: δ = 1.6 (s, NHCH_2_C*H_2_*CH_2_CH, 2H), 1.9 (s, NHCH_2_CH_2_C*H_2_*CH, 2H), 3–4.5 (m, dPG backbone, NHC*H_2_*CH_2_CH_2_CH and NHCH_2_CH_2_CH_2_C*H*NH_2_CO of arginine groups, NH_2_COC*H*CH_2_C and NH_2_COCHC*H_2_*C of histidine groups), 7.4 (s, C*H*NHCHN, 1H of imidazole groups) and 8.7 (s, CHNHC*H*N, 1H of imidazole groups) ppm.

### Gel electrophoresis

The binding of AAdPGs to siRNA was evaluated by agarose gel electrophoresis retardation assay. Stock solutions of siRNA and AAdPGs were prepared in phosphate buffer (10 mM, pH 7.4). To a 2 µL solution of siRNA (4 µM), different amounts of AAdPG compounds were added to achieve different N/P ratios (the molar ratio between amine groups of dPGs to siRNA phosphate groups). The final volume of the mixture was adjusted to 12.5 µL by the same buffer solution. siRNA and AAdPGs were incubated at room temperature for 30 min. After incubation, 2.5 µL of 6X orange gel loading dye was added to each sample. 10 µL of the final mixture was then loaded on a 1% agarose gel with 1X GelRed^TM^. After filling the gel packets with polyplexes, electrophoresis was run in TAE buffer for 45 min at 60 V. The results were visualized under UV illumination.

### DLS/Zeta

The size and zeta potential (ζ) of AAdPG/siRNA polyplexes were measured by a Zetasizer Nano ZS analyzer^TM^ with integrated 4 mW He-Ne laser, λ = 633 nm (Malvern Instruments^TM^ Ltd, U.K.). Stock solutions of dPG samples and siRNA (50 µM) in nanopure water were prepared. An appropriate amount of each dPG sample was mixed with 2.85 µL siRNA (6 nmol phosphate) solution. The mixtures were diluted to 100 µL and after short vortexing were incubated for 30 min at rt. Subsequently, DLS measurements were recorded. The same mixture from DLS measurements was taken and diluted with 0.8 µL phosphate buffer (10 mM, pH 7.4). These samples were then subjected to zeta potential measurements. The measurements were repeated at least three times for each sample and the mean values were reported.

### MTT assay

Unmodified NIH 3T3 cells were seeded at a density of 5,000 cells per well in 96-well plates 24 h in advance. The culture media was changed from 100 μL DMEM with 10% fetal bovine serum (FBS) to 80 μL plain DMEM immediately before exposure to the complexes. The dPG/siRNA complexes were prepared by first diluting the siRNA to 1.5 μM with PBS (10 mM phosphate, 10 mM NaCl, pH 7.4) and then adding the proper amount of vector solution (5 mg/mL in ddH_2_O) to give the desired N/P ratio and concentration. After 30 minutes incubation at rt, 20 μL of the complex solutions were added to each well to give a final volume of 100 μL per well. After 4 h incubation, the media was replaced with 10% FBS/DMEM and the cells cultured for another 48 h. To assess the viability, the media was replaced with 50 μL DMEM solution containing 0.5 mg/mL MTT, followed by 4 h incubation at 37 °C. 100 μL of DMSO was added to each well to dissolve the formazan and the plate was incubated at 37 °C for 30 min with agitation. The absorbance at 540 nm was measured using a plate reader and the viability determined by comparison with untreated controls.

### Transfection

NIH 3T3 fibroblast cells expressing GFP were seeded at a density of 10,000 cells/well in 48-well plates 24 h in advance and the culture media replaced with 200 μL plain DMEM immediately prior to transfection. AAdPG/siRNA complexes were prepared as described previously with either anti-GFP siRNA or negative control siRNA. 50 μL of the complex solutions were added to each well to give a final volume of 250 μL per well. After 4 h incubation, the media was replaced with 10% FBS/DMEM and the cells cultured for another 48 h. Before the analysis, cells were released from each well with trypsin and harvested by centrifugation (5 min, 500G). GFP fluorescence of transfected cells was measured on a Becton-Dickinson LSR II flow cytometer with argon ion excitation laser. For each sample, data representing 10,000 objects were collected as a list-mode file and analyzed using FACSDivaTM software (Becton Dickinson, version 6.1.3) and the percent knockdown was calculated by comparing the mean fluorescence intensity of cells treated with vector/anti-GFP siRNA to that of cells treated with complexes formed with the control siRNA.

### Cellular uptake study

For quantitative assessment of cellular uptake, negative control siRNA labeled with Cy3 (siRNA-Cy3) was complexed with the vectors in PBS as described previously. Unmodified NIH 3T3 cells were seeded in 48-well plates and transfected with the siRNA-Cy3/vector complexes following the same transfection protocol used for GFP silencing experiments. Immediately after the 4 h exposure to the transfection media, the cells were trypsinized and collected via centrifugation. The transfected cells were analyzed by FACS to determine the mean Cy3-fluoroscence of each sample.

### Confocal microscopy

Unmodified NIH 3T3 fibroblast cells were seeded at a density of 10,000 cells/well on an 8-well chamber slide (Lab-Tek, Rochester, NY) 24 h before transfection. Cy3-labeled siRNA was complexed with the vectors and the cells transfected with the complexes following the previously described protocol. After 4 h exposure to the transfection media, the media was changed back to DMEM supplemented with 10% fetal bovine serum. Confocal fluorescence spectroscopy was performed at different time points after the transfection. The nuclei were stained with Hoechst 33342 following the standard protocol. The images were acquired using a Zeiss LSM 510 inverted laser-scanning confocal microscope with a 40× numerical aperture oil immersion planapochromat objective. A 559 nm helium–neon laser, a SMD640 dichroic mirror, and a 575–620 nm band-pass barrier filter were used to obtain the images of Cy3-labeled siRNA. Images of the stained nuclei were acquired using a 780 nm two-photon excitation light, a 635 nm dichroic mirror, and a 655–755 nm band-pass barrier filter. The two fluorescent images were scanned separately and overlaid together with the differential interference contrast image (DIC). The cells were scanned as a z-stack of two-dimensional images (1024 × 1024 pixels) and an image cutting approximately through the middle of the cellular height was selected to present the intracellular siRNA localization.

### Statistical analysis

All transfection studies were performed in triplicates; data were expressed as mean ± SEM.

## Supporting Information

File 1 Synthetic procedure of dPG-NH_2_ and NMR spectra.
