# Supplementary material for: Multivalent dendritic polyglycerolamine with arginine and histidine end groups for efficient siRNA transfection
Source: Beilstein J Org Chem. 2015 May 13;11:763–72. doi: 10.3762/bjoc.11.86 (PMC4464416; doi:10.3762/bjoc.11.86)
Supplement: File 1 — Synthetic procedure of dPG-NH2 and NMR spectra. [file Beilstein_J_Org_Chem-11-763-s001.pdf]

Supporting information

for

Multivalent dendritic polyglycerolamine with arginine and histidine end groups for efficient siRNA transfection

Fatemeh Sheikhi Mehrabadi[1], Hanxiang Zeng[2], Mark Johnson[2], Cathleen Schlesener[1], Zhibin Guan [*2] and Rainer Haag[*1]

Address: [1]Institut für Chemie und Biochemie, Freie Universität Berlin, Takustrasse 3, 14195 Berlin, Germany and [2]Department of Chemistry, University of California, 1102 Natural Sciences 2, Irvine, California 92697-2025, USA

*Corresponding author

Email: Zhibin Guan - zguan@uci.edu; Rainer Haag - haag@chemie.fu-berlin.de

Synthetic procedure of dPG-NH_2 and NMR spectra

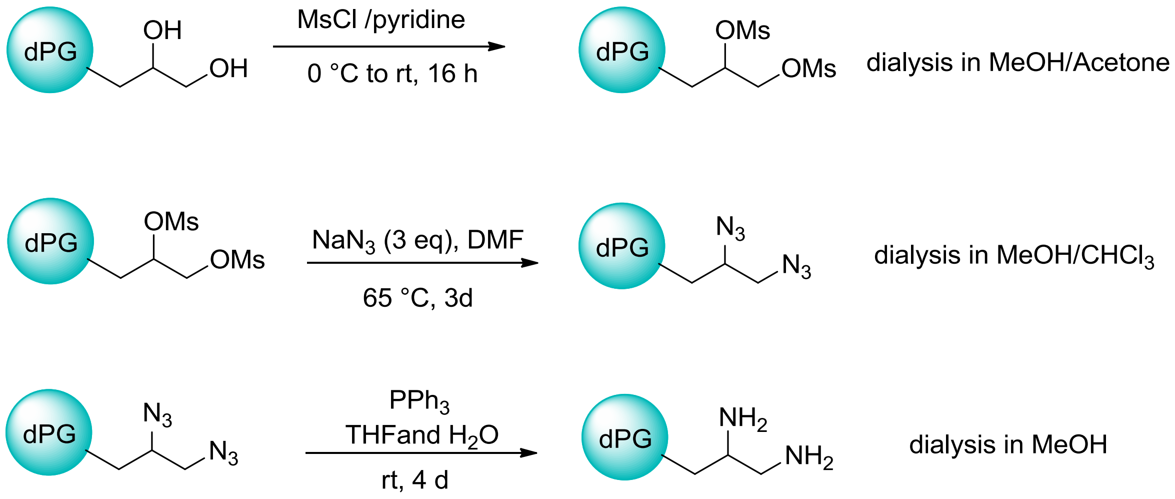

Scheme S1: Three step synthetic procedure of dPG-NH_2.

^{1}H NMR of all synthetic AAdPG vectors:

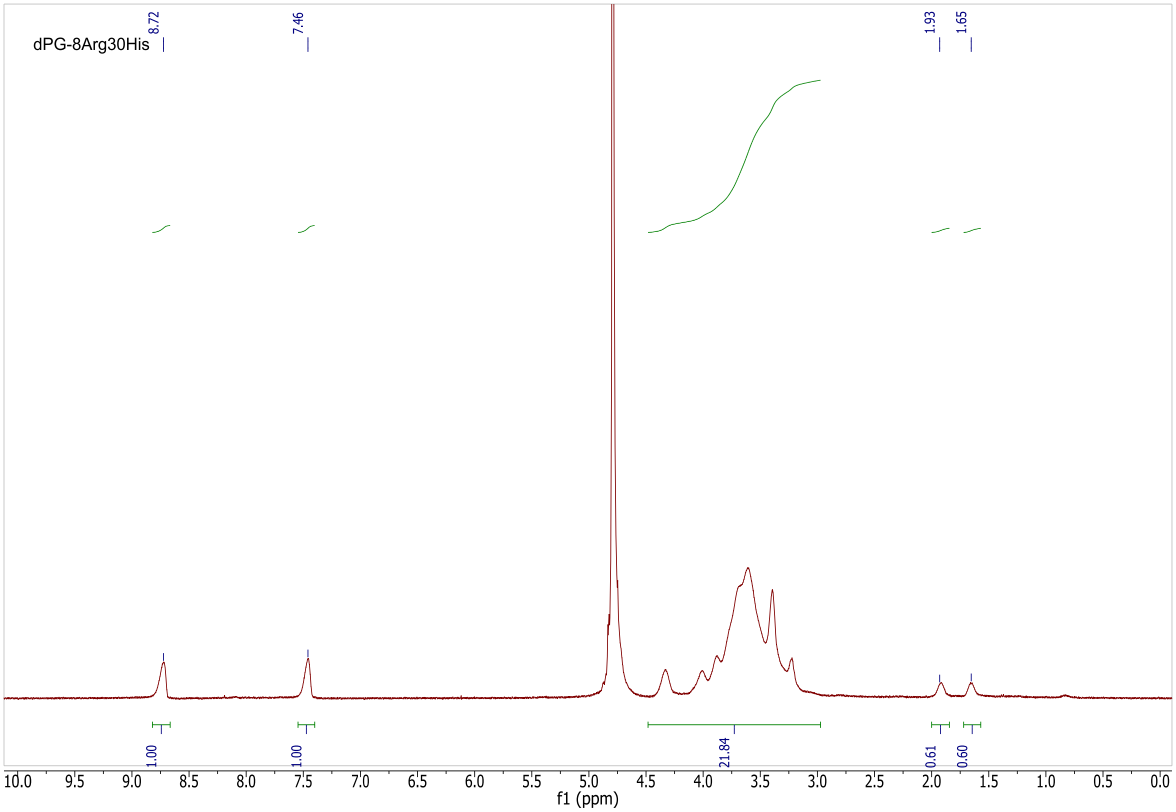

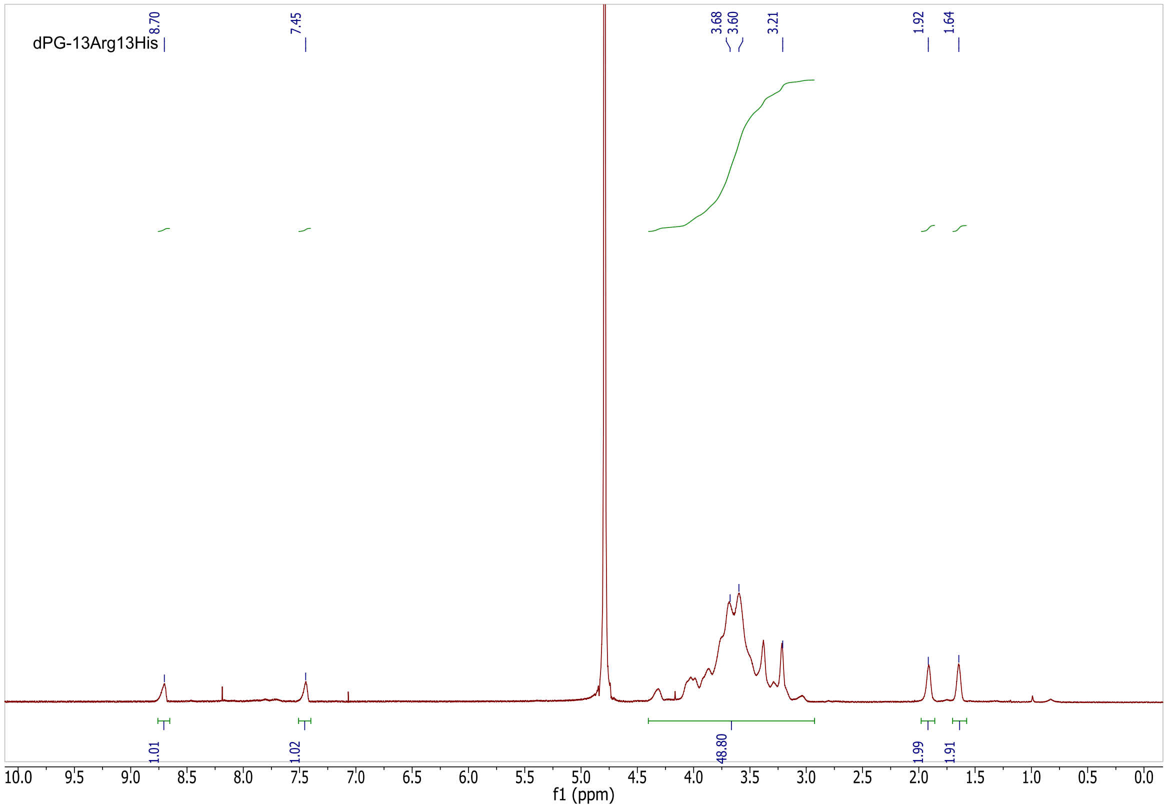

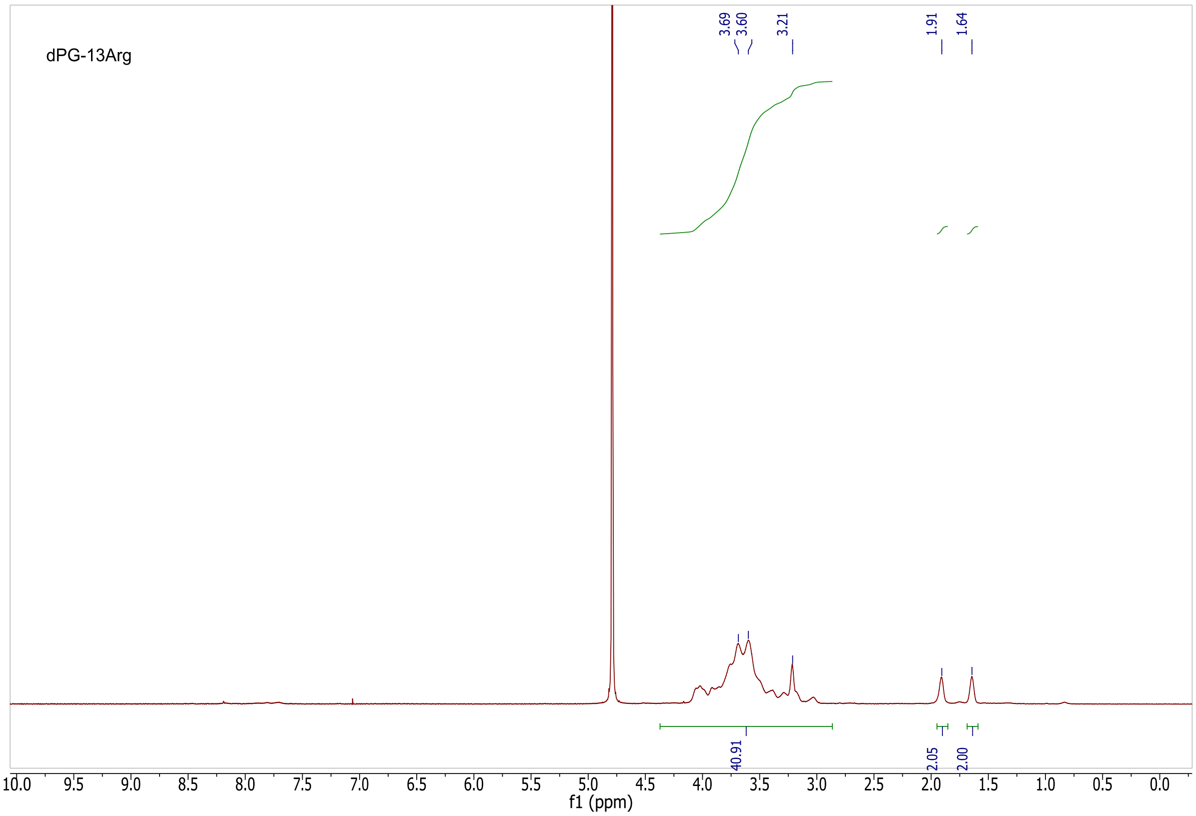
dPG-13Arg
3.69
3.60
3.21
1.91
1.64
40.91
2.05
2.00
10.0 9.5 9.0 8.5 8.0 7.5 7.0 6.5 6.0 5.5 5.0 4.5 4.0 3.5 3.0 2.5 2.0 1.5 1.0 0.5 0.0
f1 (ppm)

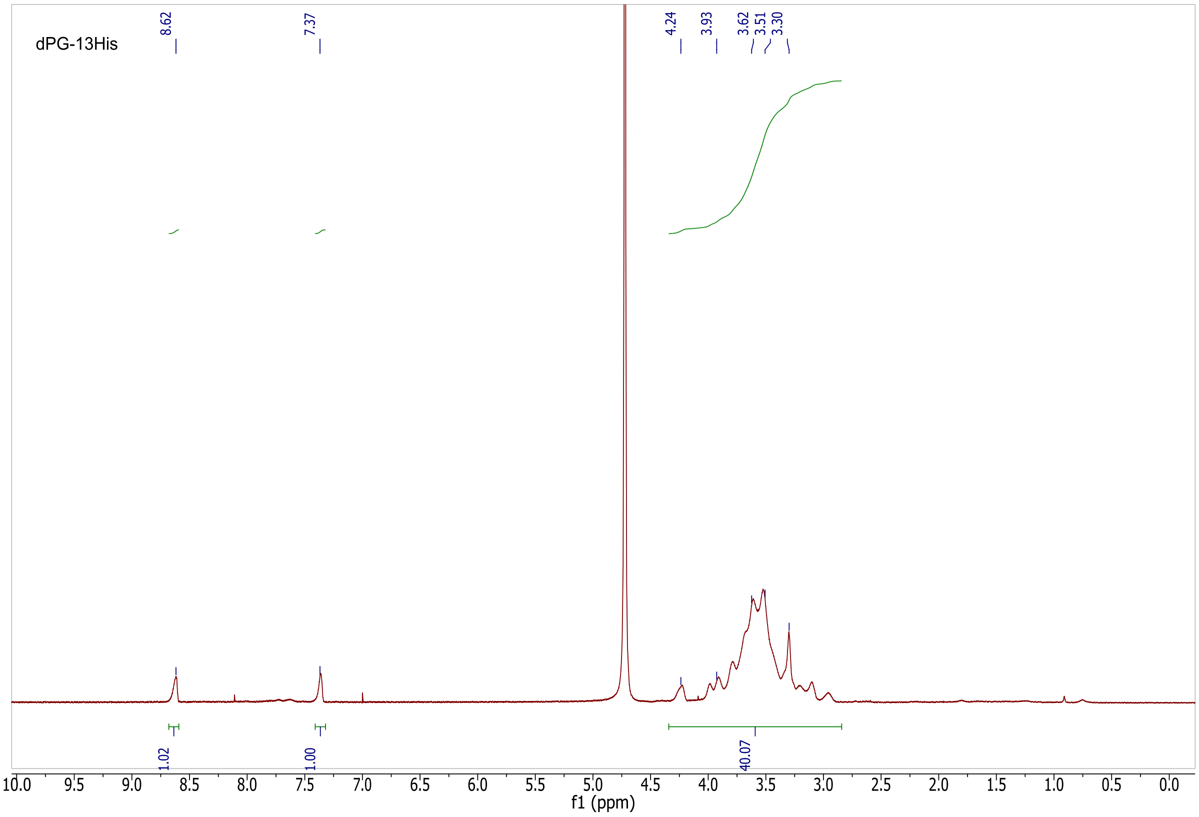
dPG-13His
8.62
7.37
4.24
3.93
3.62
3.51
3.30
1.02
1.00
40.07
10.0 9.5 9.0 8.5 8.0 7.5 7.0 6.5 6.0 5.5 5.0 4.5 4.0 3.5 3.0 2.5 2.0 1.5 1.0 0.5 0.0
f1 (ppm)
